# Parasomnia, Acute Confusion, and Transient Amnesia Temporally Associated With Ashwagandha Ingestion

**DOI:** 10.7759/cureus.95438

**Published:** 2025-10-26

**Authors:** Thushani Anuththara, Sarah S Amin, Benedict Sebastiampillai

**Affiliations:** 1 Acute Medicine, Peterborough City Hospital, Peterborough, GBR

**Keywords:** abnormal sleep, acute confusion, adverse drug reaction, ashwagandha (withania somnifera), complementary and alternative medicine, neuropsychiatric symptoms, traditional herbal medicine, transient amnesia

## Abstract

Ashwagandha (*Withania somnifera*) is a widely used adaptogenic supplement promoted for stress reduction and general wellness. Although generally well tolerated, rare but clinically significant adverse events have been documented. We report a case of acute neurotoxicity in a 40-year-old man temporally associated with ashwagandha ingestion. The patient presented with prolonged sleep, abnormal sleep behaviours, acute confusion, and retrograde amnesia. Comprehensive diagnostic evaluation excluded alternative causes. Discontinuation of ashwagandha, along with supportive management, resulted in complete symptom resolution. The underlying pathophysiology may involve GABAergic modulation mediated by withanolides, with interindividual variability in drug metabolism contributing to susceptibility. This case highlights the importance of clinician awareness regarding potential neuropsychiatric complications of herbal supplements and underscores the need for careful patient counselling about their use and possible drug interactions.

## Introduction

Ashwagandha (*Withania somnifera*), commonly known as Indian winter cherry or Indian ginseng, is a popular medicinal herb that has been extensively used in Ayurvedic medicine for thousands of years [1,2]. This evergreen shrub, belonging to the Solanaceae family, is native to India, the Middle East, southern Europe, and parts of Africa [2-4]. The Sanskrit words "ashwa" and "gandha" translate to "smell of horse", referring to the distinctive odour of its roots, while the Latin word "somnifera" signifies its "sleep-inducing" properties [1,3].

The primary bioactive compounds in ashwagandha include withanolides, alkaloids, flavonoids, and phenolic compounds, with withanolides being considered the most pharmacologically significant [1,2,5]. Clinical studies have demonstrated various potential benefits, including stress reduction, improved sleep quality, enhanced cognitive function, and anxiolytic effects [1-3]. 

In recent years, ashwagandha has gained significant popularity as a dietary supplement, particularly in Western countries, with its global market experiencing substantial growth. The herb is primarily marketed for stress and anxiety management, cognitive enhancement, and general wellness promotion [4]. The increasing consumer interest in natural adaptogens has positioned ashwagandha as one of the most widely used herbal supplements worldwide.

While ashwagandha is generally considered safe and well tolerated in clinical trials, with most studies reporting minimal adverse effects [6], emerging case reports have documented rare but serious adverse reactions. These include hepatotoxicity [2,3,7], nephrotoxicity [8], endocrine dysfunction [2,3], and various neuropsychiatric effects ranging from sedation to more severe manifestations [2,3,9,10]. The widespread use of ashwagandha supplements, often without medical supervision, has raised significant safety considerations regarding potential drug interactions [2,3,7].

Neuropsychiatric adverse effects associated with ashwagandha, while uncommon, can present significant diagnostic challenges for clinicians. We present a case of parasomnia, acute confusion, and retrograde amnesia in a previously well 40-year-old man temporally associated with ashwagandha consumption. This case highlights the potential for serious neuropsychiatric adverse effects from this widely available herbal supplement and emphasizes the importance of including herbal medications in comprehensive medication histories during the clinical evaluation of acute neurological presentations.

## Case presentation

A 40-year-old man presented to the emergency department in July 2025 with a one-day history of acute-onset confusion and altered behaviour. He had a background history of well-controlled hypertension and intermittent headaches, managed as migraine. The patient was brought in late at night by his wife, who reported that the symptoms began a few hours after he had ingested two tablets of ashwagandha, which he had taken for stress and headache relief. He had also taken one tablet the previous day without immediate adverse effects. After going to sleep that night, the patient experienced prolonged sleep lasting approximately 23 hours, with intermittent episodes of abnormal behaviour and unusual movements during sleep, as observed by his wife. He was arousable during these episodes but appeared confused and promptly returned to sleep. Video footage recorded by the patient's wife demonstrated movements that were not consistent with seizure semiology. Upon waking up the following day, he remained drowsy and disoriented, which prompted his presentation to the hospital. He was an ex-smoker and had no significant history of recreational drug use. His regular medications included ramipril and propranolol. There was no history of seizures, mental illness, or recent psychological stressors. 

**Figure 1:**
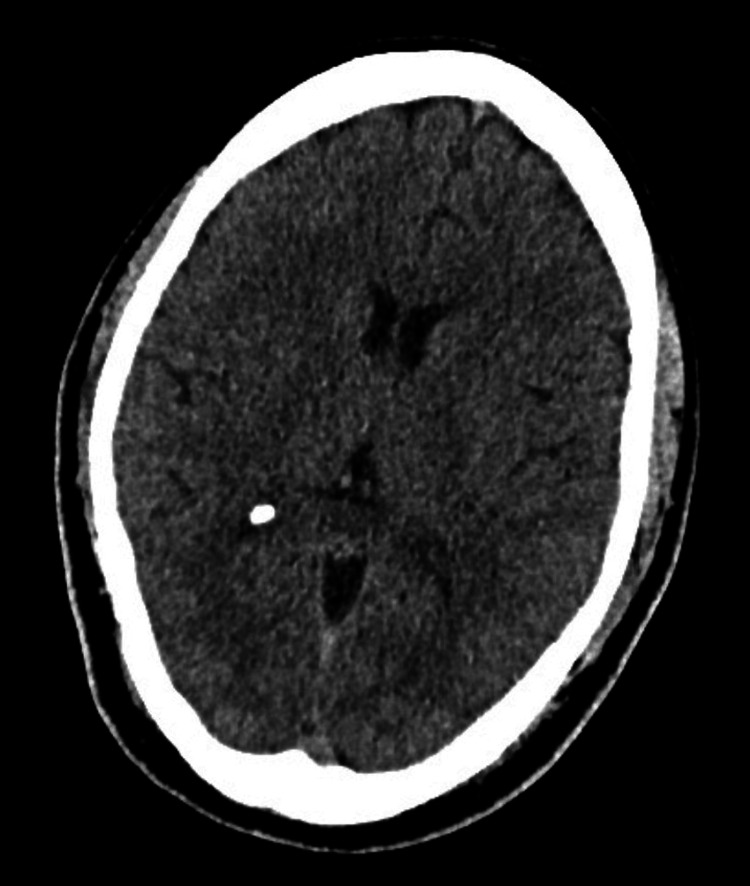
Non-contrast CT scan of the head Non-contrast CT scan of the head showing no evidence of acute intracranial pathology. Ventricular size and sulcal pattern are within normal limits, with no signs of haemorrhage, mass effect, or midline shift.

On initial assessment, the patient was haemodynamically stable with normal vital signs (blood pressure 138/89 mmHg, heart rate 91 bpm, temperature 35.8°C, respiratory rate 17/min, oxygen saturation 96% on room air), but exhibited marked confusion, with a 4 A's Test (4AT) score of 10 (Alertness: 4, Abbreviated Mental Test-4 (AMT4): 1, Attention: 1, Acute change: 4). His speech was inappropriate, with occasional incomprehensible utterances. Neurological examination revealed no focal deficits, and the remainder of the physical examination was unremarkable. Initial blood tests revealed leucocytosis (white cell count 15.8×10⁹/L) with neutrophilia (11.3×10⁹/L), while inflammatory markers remained within normal limits. An urgent CT scan of the head showed no acute intracranial pathology (Figure 1).

A summary of laboratory investigations during the hospital stay is presented in Table 1.

**Table 1:** Summary of laboratory investigations including haematological, biochemical, and immunological parameters performed during the hospital stay Findings demonstrated leucocytosis with neutrophilia, while inflammatory markers and immunological tests were within normal limits. All other values were within normal laboratory reference ranges. Chest X-ray and urine dipstick analysis were normal and showed no evidence of infection. CRP: C-reactive protein; eGFR: estimated glomerular filtration rate; ALT: alanine transaminase; ALP: alkaline phosphatase; INR: international normalized ratio; TSH: thyroid-stimulating hormone; ANA: anti-nuclear antibody; ANCA: anti-neutrophil cytoplasmic antibody; IIF: indirect immunofluorescence

Blood investigations	Result	Reference range	Unit
White blood cells	15.8	4.0-11.0	10^9^/L
Neutrophils	11.3	1.8-7.7	10^9^/L
Haemoglobin	159	130-180	g/L
Platelets	293	150-400	10^9^/L
CRP	4	<5	mg/L
Lactate	1.61	<2	mmol/L
Sodium	141	133-146	mmol/L
Potassium	4.3	3.5-5.3	mmol/L
Magnesium	0.94	0.70-1.00	mmol/L
Adjusted calcium	2.37	2.20-2.60	mmol/L
Phosphate	1.15	0.80-1.50	mmol/L
Creatinine	76	59-104	umol/L
eGFR	>90	>90	mL/min/1.73 m^2^
Urea	4.4	2.5-7.8	mmol/L
Albumin	48	35-50	g/L
Total bilirubin	17	0-21	umol/L
ALT	28	<41	IU/L
ALP	67	30-130	IU/L
Total protein	70	60-80	g/L
INR	0.98	0.8-1.25	Ratio
TSH	1.08	0.30-4.20	mU/L
Random cortisol	226	140-690	nmol/L
Vitamin B12	405	200-771	ng/L
Folate	5.6	>3.0	ug/L
Ferritin	361	30-400	ug/L
Iron	7.4	5.8-34.5	umol/L
Unsaturated iron binding capacity	65.9	22.3-61.7	umol/L
Paracetamol	14	10-30	mg/L
Salicylate	<4	15-30	mg/L
ANA	Negative	N/A	Result
ANCA (IIF)	Negative	N/A	Result

The patient was admitted with a working diagnosis of infectious encephalitis for further investigation and management. Blood culture and lumbar puncture were performed prior to initiating empirical intravenous ceftriaxone for potential bacterial meningoencephalitis and intravenous acyclovir for possible viral encephalitis. Supportive care with intravenous fluids and close neurological monitoring was initiated, and ashwagandha was discontinued immediately. 

**Figure 2:**
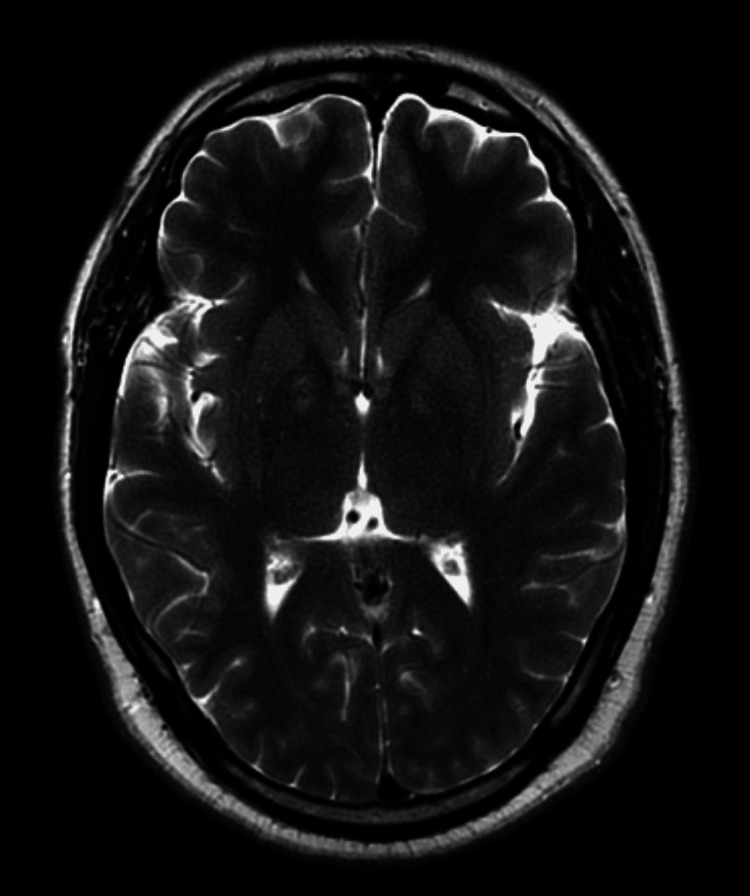
Contrast-enhanced MRI of the brain Contrast-enhanced MRI of the brain showing no evidence of intracranial pathology. There are no signal abnormalities or contrast-enhancing lesions in the temporal lobes. Ventricular size, sulcal pattern, and brain parenchyma appear within normal limits.

Approximately six hours after admission, the patient showed signs of clinical improvement, with increased coherence in speech and improved orientation (4AT score: 6; Alertness: 0, AMT4: 0, Attention: 2, Acute change: 4). However, he continued to exhibit mild psychomotor slowing and had slight difficulty following complex instructions. He remembered taking the tablets before going to bed but exhibited retrograde amnesia for events that occurred during the period of altered consciousness. Given the persistence of symptoms, an MRI of the head was arranged which did not reveal any structural abnormalities (Figure 2). An electroencephalogram (EEG) was not performed, as seizure activity was deemed clinically unlikely.

The result of the cerebrospinal fluid (CSF) analysis is presented in Table 2.

**Table 2:** CSF analysis CSF analysis demonstrating normal findings. Parameters including cell count, protein, glucose, and microbiological studies showed no evidence of infection or inflammation. CSF: cerebrospinal fluid; PCR: polymerase chain reaction; HSV: herpes simplex virus; VZV: varicella-zoster virus; DNA: deoxyribonucleic acid; RNA: ribonucleic acid

CSF analysis	Result	Reference value	Unit
CSF appearance	Clear colourless fluid	Clear colourless	Result
CSF white blood cells	6	0-5	10^6^/L
CSF red blood cells	286	0	10^6^/L
CSF protein	0.35	0.15-0.45	g/L
CSF glucose	3.4	2.2-4.0	mmol/L
Serum glucose	5.2	3.9-5.4	mmol/L
CSF Gram stain	Bacteria not seen	Bacteria not seen	Result
CSF culture	No growth	No growth	Result
HSV-1 and HSV-2 DNA PCR	Not detected	Absent	Result
VZV DNA PCR	Not detected	Absent	Result
Enterovirus RNA PCR	Not detected	Absent	Result
Parechovirus RNA PCR	Not detected	Absent	Result

Over the following days, the patient showed progressive clinical improvement, with complete resolution of symptoms. By the third day of hospitalization, he had returned to his baseline cognitive function. All antimicrobial treatments were discontinued after infectious causes were excluded. At discharge, the patient was asymptomatic, with a normal neurological examination and intact cognitive function. He was discharged home with instructions to avoid ashwagandha supplementation. At the two-week follow-up, he had successfully returned to his usual daily activities, showing sustained recovery without any residual neurological deficits. In light of the complete resolution of symptoms and absence of new concerns, further investigations were deemed unnecessary.

## Discussion

This case highlights a probable rare but concerning adverse reaction to ashwagandha (*Withania somnifera*) supplementation, characterized by prolonged sleep, abnormal behaviours during sleep, and acute confusion with retrograde amnesia upon awakening. Several factors support a causal relationship between the supplement and the patient's presentation.

The comprehensive diagnostic workup effectively excluded other common causes of acute encephalopathy. Infectious encephalitis was ruled out by normal CSF analysis and negative viral polymerase chain reaction (PCR). Autoimmune encephalitis was excluded by negative autoimmune markers. Metabolic encephalopathy was ruled out by normal electrolytes and thyroid function. Structural brain lesions were excluded by normal imaging studies. The elevated white blood cell count likely represented a stress response or mild inflammatory reaction rather than infectious pathology, given the negative microbiological studies and rapid clinical improvement.

The temporal association between *Withania somnifera *ingestion and symptom onset and rapid improvement following discontinuation, combined with the absence of prior or subsequent episodes, strongly support a drug-induced aetiology. He had recently increased his ashwagandha dosage from one (250 mg of ashwagandha extract) to two tablets (500 mg of ashwagandha extract) over two consecutive days. Hence, the absence of structural brain abnormalities or identifiable infectious, metabolic, autoimmune, or psychiatric causes raises suspicion of a possible ashwagandha-induced neuropsychiatric idiosyncratic reaction.

Furthermore, the clinical presentation is consistent with the known pharmacological properties of ashwagandha's bioactive compounds, and similar neuropsychiatric adverse events have been documented in the literature, establishing biological plausibility [11,12]. Causality assessment was performed using the Naranjo Adverse Drug Reaction Probability Scale, yielding a score of 7 (probable adverse drug reaction): temporal relationship (+2), improvement after discontinuation (+1), no alternative causes identified (+2), dose-response relationship evident (+1), and similar reactions documented in literature (+1) [13]. The World Health Organization Uppsala Monitoring Centre (WHO-UMC) causality assessment categorized this event as "probable/likely". While rechallenge was not performed for ethical and safety reasons, the cumulative evidence strongly supports a probable causal association [14]. 

There is currently no universally established daily recommended dosage for ashwagandha. Reported effective dosages in the literature vary considerably, with several sources citing doses ranging from 300 mg to 12,000 mg per day, a variation likely attributable to factors such as extract standardization, formulation, and therapeutic indication [3,15]. In this case, the patient's intake was within the recommended limits; however, he still developed significant adverse effects. This suggests that individual susceptibility factors, potentially influenced by genetic polymorphisms, may predispose certain individuals to unexpected or exaggerated responses, even at recommended doses. 

While ashwagandha is generally well tolerated and typically reported to have anxiolytic and neuroprotective properties in clinical studies [1-3,6], isolated case reports have documented neuropsychiatric adverse effects, which can present significant diagnostic challenges for clinicians. Commonly reported effects include somnolence and less commonly dizziness, vertigo, delirium, and hallucinogenic effects mimicking organic brain syndromes [2,3,9,10,16]. In clinical trials, somnolence has been reported in up to 21.1% of patients receiving ashwagandha [17]. Additionally, ashwagandha is reported to cause abnormal dream experiences, with users frequently reporting "strange and unusually vivid dreams", particularly during the first few days of supplementation, though these effects may diminish with continued use [10,18]. More serious neurological adverse effects have been documented, including acute-onset dystonia following supplementation [9] and serotonin syndrome when combined with selective serotonin reuptake inhibitors [19]. 

Ashwagandha contains various bioactive compounds, including withanolides, alkaloids, flavonoids, and phenolic compounds. Potential mechanisms underlying its neuropsychiatric effects involve the modulation of the GABAergic system, whereby compounds such as withanolides and alkaloids may enhance GABAergic neurotransmission [11,12]. This enhancement can induce sedation and, consequently, cognitive effects such as confusion and amnesia. Hence, patients concurrently using benzodiazepines, anticonvulsants, or barbiturates should exercise caution due to the risk of potentiated central nervous system depression [3,20]. While the precise mechanisms responsible for these cognitive side effects remain to be fully elucidated, individual metabolic variability arising from genetic polymorphisms in drug-metabolizing enzymes and subsequent altered interactions with neurotransmitter systems may increase susceptibility to adverse outcomes in certain individuals.

Interference with the acetylcholine pathway by *Withania somnifera* may modulate cholinergic systems involved in memory and consciousness. The plant's inhibitory effect on acetylcholinesterase (AChE) and its ability to upregulate choline acetyltransferase (ChAT) levels in rodent models have been primarily attributed to withaferin-A, a bioactive withanolide compound found in *Withania somnifera*. This dual action enhances cholinergic neurotransmission in key brain regions such as the basal ganglia and cerebral cortex, which is proposed to underlie the observed improvements in cognitive function. Furthermore, in a mouse model of Parkinson's disease, administration of *Withania somnifera *was found to suppress oxidative stress and apoptotic pathways in dopaminergic neurons, implicating a neuroprotective role within the basal ganglia, particularly the substantia nigra. Taken together, these findings, especially in light of the plant's molecular targets and AChE inhibitory activity, suggest that *Withania somnifera *may also influence dream-related cognitive phenomena, including the potential induction of lucid dreaming [21]. 

In this case, the patient exhibited abnormal movements during sleep, accompanied by confusion and amnesia upon awakening. While anecdotal reports on the internet suggest that *Withania somnifera* may induce lucid dreaming, there is a lack of evidence in the scientific literature linking ashwagandha to parasomnias. According to the International Classification of Sleep Disorders, Third Edition (ICSD-3), the clinical features observed in our patient are consistent with confusional arousals, also known as "sleep drunkenness". Confusional arousals are a form of non-rapid eye movement (NREM) parasomnia characterized by partial awakenings during slow-wave sleep (stage N3). During these episodes, individuals may display disorientation, inappropriate behaviours, and impaired responsiveness, which can persist from several minutes to hours. Notably, individuals typically experience complete amnesia for the event upon full awakening [22]. 

Several limitations of this case report warrant acknowledgment. First, as a single case report, definitive causality cannot be established, and the event may represent a rare idiosyncratic reaction rather than a class effect. Second, rechallenge was not performed for ethical and safety considerations, precluding confirmation through positive re-exposure. Third, serum levels of ashwagandha compounds or metabolites were not measured, limiting our understanding of pharmacokinetic factors. Fourth, genetic testing for polymorphisms in drug-metabolizing enzymes was not performed, which may have provided insights into individual susceptibility. Fifth, the exact composition and quality of the ashwagandha supplement consumed by the patient were not independently verified through laboratory analysis. Information regarding its constituents was obtained solely from the nutritional labelling provided by the manufacturer because, in clinical practice, laboratory analysis of dietary supplements is not routinely undertaken. Despite these limitations, the robust temporal relationship, positive dechallenge, comprehensive exclusion of alternative diagnoses, dose-response relationship, biological plausibility, and consistency with existing literature provide substantial evidence for a probable causal association.

This case highlights several important clinical considerations. Healthcare providers should routinely inquire about herbal and over-the-counter supplements when taking comprehensive medication histories. Even widely used herbal supplements can cause significant neuropsychiatric reactions in susceptible individuals, representing potential for serious adverse effects. Drug-induced encephalopathy can mimic infectious or autoimmune encephalitis, creating diagnostic challenges that require careful clinical assessment. Consumers should be informed about potential risks associated with herbal supplements through appropriate patient education.

## Conclusions

This case illustrates a rare presentation of parasomnia, acute confusion, and retrograde amnesia temporally associated with ashwagandha supplementation in an otherwise cognitively intact adult, likely mediated by the modulation of neurotransmission. The patient experienced complete symptom resolution following the discontinuation of the supplement and supportive care.

Clinicians should remain vigilant for potential adverse effects associated with herbal supplements, including those generally regarded as safe. This case emphasizes the importance of considering herbal supplements as potential causes of acute unexplained neuropsychiatric symptoms and highlights the need for comprehensive medication histories in clinical practice. Further research is warranted to better understand the mechanisms and risk factors contributing to adverse reactions from widely used herbal products such as ashwagandha.
